# Community-onset bloodstream infection with multidrug-resistant organisms: a matched case-control study

**DOI:** 10.1186/1471-2334-14-126

**Published:** 2014-03-04

**Authors:** Ching Jou Lim, Allen C Cheng, David CM Kong, Anton Y Peleg

**Affiliations:** 1 Centre for Medicine Use and Safety, Monash University, Melbourne, Victoria, Australia; 2 Department of Epidemiology and Preventive Medicine, Monash University, Melbourne, Victoria, Australia; 3 Department of Microbiology, School of Biological Sciences, Monash University, Building 76, Wellington Road, Melbourne, Victoria 3800, Australia; 4 Department of Infectious Diseases, Alfred Health, Melbourne, Victoria, Australia

**Keywords:** Multidrug-resistant, Empiric antibiotic therapy, Bacteraemia, Enterobacteriaceae, *Staphylococcus*

## Abstract

**Background:**

Multidrug-resistant (MDR) organisms have been increasingly reported at hospital admission. Recognising the magnitude, trend and predictors for MDR organisms in community-onset bloodstream infections (COBSI) is crucial for guiding empiric antibiotic prescribing.

**Methods:**

Positive blood culture isolates recovered from patients presenting to the emergency department during a ten-year period (1^st^ Jan 2002-31^st^ Dec 2011) were assessed. Trend analyses of MDR organisms were performed. Risk factors for COBSI caused by an MDR organism and predictors for 30-day mortality were also determined.

**Results:**

A total of 1721 positive blood culture isolates were identified during the study period with a yearly incidence of 30-43 isolates/10 000 ED presentations. The proportion of MDR *Escherichia coli* causing COBSI increased from 9%-26% (*P < 0.001*), whilst methicillin-resistant *Staphylococcus aureus* remained at high levels (20%-30%). A total of 360 patients were included in a matched case-control (1:1) study, and residents in long-term care facilities (adjusted odds ratio [AOR], 4.9 [95% CI, 2.1-11.6]), home wound care (AOR, 5.5 [95% CI, 1.6-18.7]), underlying immunosuppression (AOR, 3.5 [95% CI, 1.6-7.7]), recent surgery (AOR, 3.5 [95% CI, 1.1-11.6]), and exposure to antibiotics within 3 months (AOR, 5.5 [95% CI, 2.8-10.6]) were independently associated with MDR COBSI. High risk source of COBSI, age and Pitt bacteraemia score were independent predictors for 30-day mortality.

**Conclusions:**

A concerning trend in MDR organisms causing bloodstream infection from the community is occurring. Risk factors for MDR organisms have been identified to assist in empiric antibiotic prescribing for those presenting to hospital with sepsis.

## Background

Antimicrobial resistance in common bacterial pathogens causing community-onset infections, including bloodstream infection (BSI), has increasingly been reported, most notably methicillin-resistant *Staphylococcus aureus* (MRSA) and various multidrug-resistant (MDR) gram-negative bacilli (GNB) [1,2]. This poses significant challenges to clinicians caring for patients presenting to hospital with suspected sepsis as empiric antibiotic prescribing is often targeting presumed, antibiotic-susceptible, community organisms. Identification of risk factors for MDR organisms in patients presenting from the community with sepsis is necessary to help optimise patient outcomes and minimise the use of broad-spectrum antibiotics.

Emergency departments (EDs) serve as an important interface between community and hospital care; the microbial ecology is often a combination of community-and healthcare-associated organisms, the latter being driven by healthcare re-admissions. Friedman et al. proposed that healthcare-associated (HCA) bloodstream infection should be treated with broad-spectrum antibiotics to cover for MDR organisms [3]. However, the broad nature of the HCA risk factors may lead to unnecessary use of last-line antibiotics, especially in settings with a low prevalence of MDR organisms [4]. Several studies have worked towards refining predictors for MDR organisms in community-onset pneumonia [5-7]; similar work to predict MDR organisms in community-onset bloodstream infections (COBSI) remains scant.

To date, the prevalence and trend of major MDR organisms causing COBSI remain poorly understood. Furthermore, data on risk factors and outcomes associated with COBSI due to MDR organisms are limited. Accordingly, we explored the longitudinal trend, risk factors and clinical outcomes associated with MDR organisms causing COBSI in a tertiary care institution. These data are critical in devising the most effective empiric antibiotics to maximise patient outcomes.

## Methods

### Study design and population

We performed an observational series and a matched case-control study at The Alfred, a tertiary referral hospital in metropolitan Melbourne, Australia. We studied all cases of bloodstream infection presenting from the community to The Alfred’s ED. This ED provides specialised state-wide services for major emergency and trauma, burns, heart and lung transplantation, cystic fibrosis and neurosurgery patients. It attends to over 55 000 patients annually. The study was approved by the human research ethics committees of The Alfred and Monash University.

### Data collection and definitions

Data collection was broadly divided into two parts:

i) Prevalence and temporal trends of MDR organisms

Positive clinical isolates recovered from blood cultures of patients within 48 hours of ED admission during a ten-year period (1^st^ January 2002 and 31^st^ December 2011) were examined from a microbiology laboratory database. Repeat cultures from an individual patient growing the same organism within 14 days, cultures reported as normal flora or probable contaminants (eg. coagulase-negative staphylococci from a single blood culture bottle), and clinical specimens from patients aged < 18 years, were excluded. Species identification and antimicrobial susceptibility testing were performed using an automated system (Vitek®; bioMérieux). For the majority of the study period (Jan 2002-Jun 2010), susceptibility breakpoints were based on the British Society for Antimicrobial Chemotherapy (BSAC) guidelines [8]. These were changed to the European Committee on Antimicrobial Susceptibility Testing (EUCAST) guidelines from July of 2010 [9]. We observed no change in the proportion of resistant isolates at the time of the change in breakpoint definitions.

The temporal trends of MDR GNB, MRSA, and vancomycin-resistant enterococci (VRE) were assessed. MDR was defined as being non-susceptible to at least three different classes of antimicrobial agents, as described elsewhere [10,11]. For Enterobacteriaceae, seven classes of antimicrobials were assessed, (i) extended-spectrum penicillins or first-generation cephalosporins, (ii) β-lactam/β-lactamase inhibitor combinations, (iii) extended-spectrum cephalosporins, (iv) carbapenems, (v) trimethoprim/sulfamethoxazole, (vi) aminoglycosides, and (vii) quinolones. For non-fermenting GNB, antimicrobials were divided into five classes, (i) antipseudomonal penicillins, (ii) antipseudomonal cephalosporins, (iii) carbapenems, (iv) aminoglycosides, and (v) quinolones.

Multi-resistant MRSA was defined as MRSA resistant to three or more of the following antibiotics: erythromycin, ciprofloxacin, gentamicin, trimethoprim/sulfamethoxazole, tetracycline, rifampicin, fusidic acid and vancomycin [12]. The EMRSA-15-like strain was defined as MRSA resistant to ciprofloxacin +/- erythromycin only [12]. All other MRSA were non-multi-resistant MRSA. For the *Streptococcus pneumoniae* strains, the susceptibility interpretation was based on non-central nervous system breakpoints.

ii) Risk factors and outcomes associated with MDR COBSI

A matched case-control study was subsequently conducted for risk factor analysis. A case was defined as an adult (≥ 18 years) with COBSI (organism isolated from ≥ 1 blood culture within 48 hours of ED admission) due to a MDR organism. Only the first admission with COBSI due to a MDR organism was included in the analysis. Control patients were adults with COBSI caused by non-MDR organisms matched for the genus of the causative organism and the date of ED admission (± 1 year). If more than one control was identified, the control patient with the ED admission date closest to the case patient was chosen. Cases of polymicrobial bacteraemia, and patients who were directly transferred from other hospitals with a hospital stay > 48 hours were excluded.

Information on patient demographics, sources of BSI, co-morbidities, recent bacterial infection and antibiotic use, invasive instrumentation in the preceding 48 hours, recent surgery, underlying immunosuppression, and the presence of healthcare-associated risk factors were reviewed. Sources of BSI were divided into low risk sources including urinary, skin and soft-tissue, and catheter-related, and high risk sources including respiratory, intra-abdominal, bone and joint, cardiovascular or unidentified [13]. Co-morbidities were quantified using the Charlson comorbidity index [14]. Presence of underlying immunosuppression was defined as the presence of neutropenia (absolute neutrophil count of < 500/mm^3^), concurrent use of high dose oral corticosteroids (at least 10 mg of prednisolone or equivalent for minimum of 30 days) or long-term use of immunosuppressive agents (e.g. cyclosporine), active chemotherapy for malignancy, metastatic cancer, or acquired immunodeficiency syndrome. Healthcare-associated (HCA) BSI was defined according to Friedman et al. [3]. This included patients who had been hospitalised in an acute care hospital for ≥ 2 days in the preceding 90 days, residents of a nursing home or long-term care facility (LTCF), patients who received home intravenous (IV) therapy, specialised wound care, and those who underwent chronic haemodialysis or IV chemotherapy in the preceding 30 days.

Information related to clinical presentation, management and outcomes of BSI was also collected, including severity of BSI according to the Pitt bacteraemia score [points assigned to specific clinical observations as (1) oral temperature of 35.1°C-36.0°C or 39.0°C-39.9°C scores 1 point, and a temperature of either ≤35°C or ≥40°C scores 2 points; (2) systolic blood pressure < 90 mm Hg for >1 hour despite adequate fluid resuscitation, receipt of vasopressors, and/or receipt of mechanical ventilation score 2 points each; (3) cardiac arrest scores 4 points; and (4) altered mental status scores 1 point for disorientation, 2 points for stupor, and 4 points for comatose] [1], presence of severe sepsis or septic shock, types and duration of empiric antibiotic therapy, length of hospital stay and mortality within 30 days of COBSI. Empirical antibiotic therapy was considered active when an antibiotic was given during the first 48 hours that had in vitro activity against the causative organism.

### Statistical analysis

Microbiological data were available from 2002 and denominator data on the number of ED presentations per year were available from 2004 onwards. A rate was calculated based on the number of isolates per 10 000 ED presentations per year. Bi-yearly trends in distribution of major organisms and antimicrobial resistance patterns were assessed using the chi-squared test for trend or linear regression.

Risk factor analysis was performed using conditional logistic regression analyses, firstly by univariate, followed by multivariate analysis. Variables with a *P <* 0.1 on univariate analysis were eligible for entry into the multivariable model. The variables of ‘invasive instrument in-situ’, ‘underlying immunosuppression’ and ‘prior exposure to antibiotic’ were considered for inclusion in the multivariable model as binary variables, ignoring constituent variables within the categories. For correlated variables (pairwise correlation coefficient > 0.7), only one of the covariates was selected for inclusion into the candidate models on the basis of the strength of association. Independent variables in the final multivariable model were selected using a stepwise forward process. Model fit was assessed against the number of variables used, quantified using the Akaike information criterion to prevent overfitting. Risk factor analysis for COBSI caused by any MDR organism was performed first followed by a risk factor analysis for specific MDR organisms (MDR GNB and MRSA).

In addition, categorical and continuous variables associated with 30-day all cause mortality were compared between the survival and non-survival groups using univariate and then multivariate logistic regression analyses. All tests were 2-tailed, and *P-*values of *<* 0.05 were considered statistically significant. Data analyses were performed using IBM SPSS Statistics version 20 (IBM, New York, USA).

## Results

### Temporal trend of MDR organisms causing COBSI

A total of 1721 positive blood culture isolates were identified over a 10-year period from patients presenting to the ED. From 2004 onwards, there was a yearly incidence of 30-43 BSI per 10 000 ED presentations (Table 1). Overall, gram-negative organisms (n = 941, 55%) predominated (Table 1). When adjusted for the number of ED presentations over time, the yearly incidence rate of gram-positive isolates declined over time (*P* = 0.02 for trend), whilst gram-negative isolates remained relatively constant (*P* > 0.05 for trend).

**Table 1:** Yearly incidence of positive blood culture isolates, comparing gram-positive with gram-negative isolates

**Year**	**Number of ED presentations**	**Yearly incidence of positive blood culture isolates**					
		**Total blood culture isolates, number**	**Total blood culture isolates per 10 000 ED presentation**	**Gram-negative isolates, number**	**Number of gram-negative isolates per 10 000 ED presentation**	**Gram-positive isolates, number**	**Number of gram-positive isolates per 10 000 ED presentation**
2002	-	156	-	67	-	89	-
2003	-	199	-	107	-	92	-
2004	37765	152	40.3	81	21.5	71	18.8
2005	41912	153	36.5	84	20.0	69	16.5
2006	44417	189	42.6	109	24.5	80	18.0
2007	46979	192	40.9	100	21.3	92	19.6
2008	47356	183	38.6	103	21.8	80	16.9
2009	50155	156	31.1	90	17.9	66	13.2
2010	52985	161	30.4	97	18.3	64	12.1
2011	56120	180	32.1	103	18.4	77	13.7
**Total**		1721		941		780	

NOTE. Data on the number of ED presentations was available from 2004.

**Figure 1:**
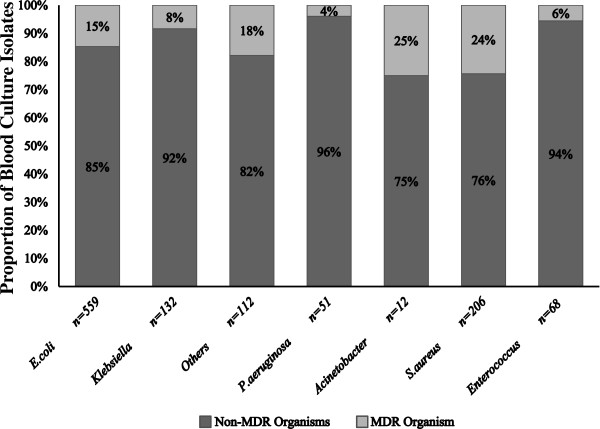
**The proportion of MDR organisms versus non-MDR organisms in positive blood culture isolates, according to genus.** ‘*Others*’ in the figure refers to other Enterobacteriaceae with ≥ 10 blood isolates during study period (ie. *Proteus, Enterobacter, Citrobacter* and *Serratia* spp.). MDR organisms in *S. aureus* refers to MRSA whilst in *Enterococcus* refers to VRE.

The proportion of MDR organisms according to genus is shown in Figure 1. Overall, *Escherichia coli* was the most common causative gram-negative organism. The proportion of MDR *E. coli* (n = 82) increased significantly during the study period from 9% (2002-2003) to 26% (2010-2011) (*P* < 0.001 for trend). Increasing resistance to important first-line agents was also observed from years 2002-2003 to 2010-2011; ceftriaxone (1% to 6%, *P* < 0.01), gentamicin (1% to 12%, *P* < 0.001) and ciprofloxacin (3% to 22%, *P* < 0.01). In contrast, the incidence of MDR *Klebsiella* spp. (n = 11) and other Enterobacteriaceae (n = 20) did not show a significant increase over time; however this may be partly explained by the small number of isolates. As expected, BSI caused by non-fermenting GNB were less common; however there were still 51 and 12 cases of *Pseudomonas aeruginosa* and *Acinetobacter* spp. in COBSI, respectively. MDR cases were also observed (2/51, [4%] for *P. aeruginosa* and 3/12, [25%] for *Acinetobacter* spp.).

*S. aureus* was the most common gram-positive organism causing COBSI (n = 206, 26%), with a stable yet high proportion of MRSA over time of 20%-30%. The majority of MRSA were of a multi-resistant phenotype (n = 41 [57%]), followed by EMRSA-15-like strains (n = 16 [22%]) and then non-multi-resistant strains (n = 15 [21%]). Over the 10 years, four VRE in COBSI were identified. About 10% of *Streptococcus pneumoniae* blood culture isolates (n = 104) were non-susceptible to penicillin; none of these were resistant to ceftriaxone.

### Risk factors for COBSI due to MDR organisms

There were 194 MDR blood culture isolates identified from 2002-2011. Fourteen isolates were excluded; polymicrobial BSI (n = 7), multiple BSI episodes in the same patient (n = 3), direct transfer from other hospital (n = 1) and incomplete/missing medical histories (n = 3). Accordingly, 180 case patients were included, with 109 MDR GNB (comprised of *E. coli* [n = 79], *Klebsiella* spp. [n = 8], other Enterobacteriaceae [n = 17], *P. aeruginosa* [n = 2] and *Acinetobacter* spp. [n = 3]) and 67 MRSA in the subgroup analyses.

The characteristics of case and control patients are shown in Table 2. Sources of COBSI due to MDR organisms composed primarily of urinary sites (n = 60, 33%) and unidentified sources (n = 53, 29%), followed by catheter-related (n = 18, 10%), skin and soft tissue (n = 16, 9%), bone and joint (n = 13, 7%), intra-abdominal (n = 10, 6%), and respiratory sites (n = 10, 6%). Overall, 82% of the case patients met the Friedman et al. criteria [3] for HCA BSI, however 40% of the control patients also had at least one of these defined risk factors. Multivariable analysis showed that residents in LTCF, home wound care, underlying immunosuppression, recent surgery and prior exposure to antibiotics were independent risk factors for COBSI caused by a MDR organism (Table 2).

**Table 2:** Patient characteristics and risk factor analysis for community-onset bloodstream infection caused by a MDR organism

**Risk factors**	**Cases**	**Controls**	**Univariate analysis**	**Multivariate analysis**		
	**(n = 180)**	**(n = 180)**	**OR (95% CI)**	***P value***	**AOR (95% CI)**	***P value***
Age, mean (SD), years	64.1 (19.4)	65.0 (20.4)	1.00 (0.99-1.01)	0.7		
Female, no (%)	69 (38.3)	74 (41.1)	0.94 (0.75-1.18)	0.59		
Source of BSI, low risk^b^, no (%)	94 (52.2)	83 (46.1)	1.32 (0.85-2.03)	0.22		
Invasive device in-situ, no (%)	55 (30.6)	21 (11.7)	3.62 (1.96-6.68)	<0.001^a^		
Indwelling catheter	25 (13.9)	8 (4.4)	3.43 (1.48-7.96)	<0.01		
Vascular catheter	27 (15.0)	12 (6.7)	2.67 (1.24-5.74)	0.01		
Percutaneous feeding tube	7 (3.9)	1 (0.6)	7.00 (0.86-56.89)	0.07		
Friedman et al. HCA risk factors, no (%)	148 (82.2)	72 (40.0)	6.81 (3.81-12.19)	<0.001		
Prior hospitalisation (≥2d) last 90d	113 (62.8)	44 (24.4)	4.62 (2.81-7.58)	<0.001^a^		
Residence in long-term care facilities	51 (28.3)	23 (12.8)	2.79 (1.57-4.94)	<0.001^a^	4.94 (2.11-11.59)	<0.001
Home intravenous therapy last 30d	27 (15.0)	9 (5.0)	2.89 (1.35-6.17)	0.006^a^		
Home wound care last 30d	35 (19.4)	5 (2.8)	6.96 (2.72-17.80)	<0.001^a^	5.48 (1.61-18.69)	<0.01
Haemodialysis last 30d	5 (2.8)	12 (6.7)	0.42 (0.15-1.18)	0.100		
Intravenous chemotherapy last 30d	32 (17.8)	15 (8.3)	2.31 (1.20-4.42)	0.012^a^		
Underlying immunosuppression, no (%)	77 (42.8)	35 (19.4)	3.73 (2.11-6.60)	<0.001^a^	3.49 (1.58-7.71)	<0.01
Neutropenia	23 (12.8)	11 (6.1)	2.20 (1.04-4.65)	0.04		
Active or metastatic cancer	45 (25.0)	25 (13.9)	2.11 (1.21-3.70)	0.01		
Concurrent immunosuppressant agents	31 (17.2)	8 (4.4)	4.14 (1.82-9.46)	<0.01		
Surgery last 30d, no (%)	38 (21.1)	9 (5.0)	6.60 (2.58-16.91)	<0.001^a^	3.52 (1.06-11.64)	0.04
Bacterial infection last 3mo, no (%)	99 (55.0)	34 (18.9)	5.32 (3.06-9.23)	<0.001^c^		
Presence of BSI last 3mo, no (%)	36 (20.0)	7 (3.9)	6.80 (2.66-17.39)	<0.001^a^		
Prior antibiotic exposure last 3mo, no (%)	127 (70.6)	39 (21.7)	7.73 (4.34-13.78)	<0.001^a^	5.49 (2.84-10.61)	<0.001
Prolonged exposure >14d	78 (43.3)	27 (15.0)	4.38 (2.50-7.68)	<0.001		
Fluoroquinolone	43 (23.9)	9 (5.0)	5.86 (2.63-13.06)	<0.001		
Extended-spectrum cephalosporin	32 (17.8)	5 (2.8)	7.95 (2.80-22.55)	<0.001		
Penicillin + beta lactamase inhibitor	44 (24.4)	15 (8.3)	3.64 (1.87-7.09)	<0.001		
Vancomycin	27 (15.0)	10 (5.6)	2.78 (1.30-5.95)	0.01		
Diabetic mellitus, no (%)	45 (25.0)	28 (15.6)	1.95 (1.12-3.39)	0.018^a^		
Cardiovascular disease, no (%)	50 (27.8)	40 (22.2)	1.36 (0.85-2.16)	0.200		
Cerebrovascular disease, no (%)	30 (16.7)	27 (15.0)	1.10 (0.63-1.94)	0.74		
Lung disease, no (%)	47 (26.1)	32 (17.8)	1.66 (0.97-2.82)	0.062^a^		
Renal disease, no (%)	25 (13.9)	28 (15.6)	0.89 (0.51-1.54)	0.68		
Liver disease, no (%)	21 (11.7)	18 (10.0)	1.20 (0.61-2.38)	0.6		
Malignancy, no (%)	71 (39.4)	48 (26.7)	1.71 (1.10-2.66)	0.018^a^		
Charlson co-morbidity index, mean (SD)	3.4 (2.6)	2.0 (2.0)	1.24 (1.13-1.36)	<0.001^a^		

NOTE. OR, odds ratio; AOR, adjusted odds ratio; CI, confidence interval; SD, standard deviation; BSI, bloodstream infections; HCA, healthcare-associated.

^a^Variables eligible for inclusion in the multivariate analysis.

^b^Low risk sites are BSI secondary to urinary, skin & soft tissue and catheter-related sites.

^c^Pairwise correlation >0.7 with prior antibiotic exposure last 3mo.

Subgroup analyses for specific risk factors for MDRGNB and MRSA are shown in Table 3. In addition to being a resident in a LTCF and prior exposure to antibiotics, home IV therapy and younger age were also independently associated with COBSI due to MDR GNB. Seven of 18 (39%) MDR GNB case patients with an age < 40 years had no identified predisposing risk factors and all but one were COBSI associated with a urinary source. On the other hand, hospitalisation in the last three months, residing in LTCF, receiving home wound care and underlying immunosuppression were independently associated with COBSI caused by MRSA. A significantly higher proportion of COBSI cases with MRSA (63/67, 94%) compared to cases with MDR GNB (81/109, 74%) fulfilled at least one Friedman et al. criteria (*P* = 0.001).

**Table 3:** Subgroup analyses of risk factors for community-onset bloodstream infection caused by MDR GNB and MRSA

**Variables**	**BSI with MDR GNB, case = 109, control = 109**				**BSI with MRSA, case = 67, control = 67**			
	**Univariate analysis**		**Multivariate analysis**		**Univariate analysis**		**Multivariate analysis**	
	**OR (95% CI)**	***P value***	**AOR (95% CI)**	***P value***	**OR (95% CI)**	***P value***	**AOR (95% CI)**	***P value***
Age, years	0.99 (0.97-1.00)	0.045^a^	0.98 (0.96-1.00)	0.03	1.02 (1.00-1.04)	0.061^a^		
Female	1.05 (0.58-1.91)	0.88			0.66 (0.31-1.41)	0.29		
Low risk BSI sources^b^	1.29 (0.73-2.27)	0.39			1.71 (0.83-3.49)	0.15		
Invasive device in-situ	3.43 (1.48-7.96)	0.004^a^			5.50 (1.90-15.96)	0.002^a^		
Indwelling catheter	2.60 (0.93-7.29)	0.07			11.00 (1.42-85.20)	0.02		
Vascular catheter	3.25 (1.06-9.97)	0.04			2.50 (0.78-7.97)	0.12		
Friedman et al. HCA risk factors	4.90 (2.48-9.67)	<0.001			13.17 (4.07-42.59)	<0.001		
Prior hospitalisation (≥2d) last 90d	4.50 (2.27-8.93)	<0.001^a^			4.75 (2.31-9.75)	<0.001^a^	5.87 (1.37-25.21)	0.02
Residence in long-term care facilities	2.44 (1.13-5.31)	0.024^a^	5.56 (1.60-19.34)	<0.01	3.78 (1.53-9.33)	0.004^a^	12.38 (2.45-62.63)	<0.01
Home intravenous last 30d	8.00 (1.84-34.79)	0.006^a^	8.33 (1.52-45.75)	0.02	1.67 (0.61-4.59)	0.32		
Home wound care last 30d	1.67 (0.40-6.97)	0.48			13.94 (3.31-58.68)	<0.001^a^	6.74 (1.06-42.70)	0.04
Haemodialysis last 30d	1.50 (0.25-8.98)	0.66			0.22 (0.05-1.03)	0.054^a^		
Intravenous chemotherapy last 30d	2.86 (1.29-6.43)	0.010^a^			1.75 (0.51-5.98)	0.37		
Underlying immunosuppression	3.09 (1.57-6.10)	0.001^a^			6.67 (1.98-22.44)	0.002^a^	7.21 (1.20-43.43)	0.03
Neutropenia	2.57 (1.07-6.16)	0.03			2.00 (0.37-10.92)	0.42		
Active or metastatic cancer	2.25 (1.14-4.44)	0.02			1.80 (0.60-5.37)	0.29		
Surgery last 30d	6.50 (1.47-28.80)	0.014^a^			9.50 (2.21-40.79)	0.002^a^		
Presence of BSI last 3mo	6.00 (1.77-20.37)	0.004^a^			6.50 (1.47-28.80)	0.014^a^		
Prior antibiotic exposure last 3mo	8.29 (3.78-18.15)	<0.001^a^	7.94 (3.36-18.80)	<0.001	6.76 (2.87-15.92)	<0.001^a^		
Diabetic	1.91 (0.92-3.96)	0.082^a^			1.75 (0.73-4.17)	0.21		
Malignancy	1.81 (1.06-3.08)	0.029^a^			1.30 (0.57-2.97)	0.53		
Charlson co-morbidity index	1.16 (1.04-1.30)	0.007^a^			1.38 (1.15-1.67)	0.001^a^		
Intravenous drug user, IVDU	NA	NA			0.07 (0.01-0.51)	0.009^a^		

NOTE. OR, odds ratio; AOR, adjusted odds ratio; CI, confidence interval; BSI, bloodstream infections; HCA, healthcare-associated; NA, not applicable.

^a^Variables eligible for inclusion in the multivariate analysis.

^b^Low risk sites are BSI secondary to urinary, skin & soft tissue and catheter-related sites.

### Clinical management and outcomes of COBSI

Community-onset BSI caused by MDR organisms was significantly more likely to be treated with inactive empirical antibiotic therapy (42% vs. 1%, *P* < 0.001). The median length of hospital stay for those infected with MDR organisms was 11 days (interquartile range [IQR] 6-20) compared with 9 days (IQR 5-17) (*P* < 0.05) for those with non-MDR organisms. The overall 30-day mortality observed in both groups was 13% (46 / 360), with 16% (n = 28) in those infected with MDR organisms as opposed to 10% (n = 18) in non-MDR infections (*P* = 0.127). The prognostic factors for 30-day mortality are shown in Table 4. Older age, higher Pitt bacteraemia score, and high risk sources of bacteraemia were independent predictors for mortality within 30 days of COBSI. Community-onset BSI caused by a MDR organism, receipt of inactive empiric antibiotic therapy and COBSI defined as HCA BSI were not significant predictors.

**Table 4:** Predictors of 30-day all-cause mortality among patients with community-onset bloodstream infection

**Variable**	**Patients who died in 30 days**	**Patients who survived**	**Univariate analysis**		**Multivariate analysis**	
	**n = 46**	**n = 314**	**OR (95% CI)**	***P value***	**AOR (95% CI)**	***P value***
Age, mean (SD), years	74.8 (16.3)	63.1 (19.9)	1.04 (1.02-1.06)	<0.001^a^	1.04 (1.02-1.06)	<0.001
Male, no (%)	31 (67.4)	186 (59.2)	1.42 (0.74-2.74)	0.29		
Residence in LTCF, no (%)	18 (39.1)	56 (17.8)	2.96 (1.53-5.72)	0.001^a^		
Charlson co-morbidity index, mean (SD)	3.5 (2.2)	2.6 (2.4)	1.15 (1.02-1.30)	0.018^a^		
Acquisition-healthcare associated, no (%)	35 (76.1)	185 (58.9)	2.22 (1.09-4.53)	0.029^a^		
Underlying immunosuppression, no (%)	14 (30.4)	98 (31.2)	0.96 (0.49-1.89)	0.92		
High risk BSI sources^b^, no (%)	30 (65.2)	153 (48.7)	1.97 (1.03-3.76)	0.039^a^	2.30 (1.16-4.57)	0.02
Pitt bacteraemia score, median (IQR)	2 (1-4)	1 (0-2)	1.39 (1.18-1.64)	<0.001^a^	1.36 (1.15-1.61)	<0.001
Severe sepsis or septic shock, no (%)	13 (28.3)	38(12.1)	2.86 (1.39-5.91)	0.005^c^		
ICU admission, no (%)	12 (26.1)	51 (16.2)	1.82 (0.88-3.75)	0.11		
Bacteraemia due to MDR organisms, no (%)	28 (60.9)	152 (48.4)	1.66 (0.88-3.12)	0.12		
Inactive empiric antibiotic, no (%)	16 (34.8)	61 (19.4)	2.21 (1.13-4.31)	0.02^a^		

NOTE. OR, odds ratio; AOR, adjusted odds ratio; CI, confidence interval; SD, standard deviation; IQR, interquartile range; LTCF, long-term care facility; BSI, bloodstream infections; ICU, intensive care unit; MDR, multidrug-resistant.

^a^Variables eligible for inclusion in the multivariate analysis.

^b^High risk sources refer to BSI secondary to respiratory, gastrointestinal, hepatobiliary, bone and joint, cardiovascular or unidentified sources.

^c^Pairwise correlation >0.7 with Pitt bacteraemia score.

## Discussion

This study has shown that MDR GNB have joined MRSA as an important cause of bloodstream infection presenting from the community, highlighting the evolving challenges of empiric antibiotic prescribing for severe infections at the front-line of hospital admission. We identified differences in risk factors for MDR GNB and MRSA that are in keeping with the changing epidemiology of these organisms. The increase in MDR GNB was largely attributable to MDR *E. coli*, whereas MDR in non-fermenting GNB remained relatively uncommon. Traditional risk factors for infection with an MDR organism such as HCA risk factors proposed by Friedman et al. [3], appeared predictive for MRSA COBSI but were less predictive for MDR GNB infection. Patients with COBSI due to an MDR organism were more likely to be prescribed inactive empiric therapy and were associated with longer hospital stays; nonetheless, COBSI with an MDR organism was not related to significantly higher 30-day mortality.

In the last decade, emergence of MDR GNB at hospital admission has been reported [1,15,16]. The most recent Australian surveillance data revealed an emerging trend of MDR *E. coli* in urinary isolates from the community, rising from 4.5% in 2008 to 7.2% in 2010 [17]. Apart from the known increase in MDR GNB causing community urinary tract infections, fewer studies have focused on the trends in more severe community infections like bloodstream infection [1]. Our data showed that a staggering 26% of *E. coli* bloodstream infection presenting to ED was MDR. Our standard first-line empiric therapy to cover GNB for suspected sepsis is often an aminoglycoside or a third-generation cephalosporin [18]; however 6% and 12% of blood culture isolates were resistant to these agents, respectively. Furthermore, 22% were resistant to ciprofloxacin. Identifying risk factors to help determine who should receive broader-spectrum antibiotics for patients presenting to ED with suspected BSI is needed.

In contrast to previous studies [16,19], older age was not shown to be a predictor for acquiring MDR organisms; on the contrary, older patients were less likely to have a COBSI with MDR GNB. Our data suggest a potential shift in the epidemiology of MDR GNB to a younger patient population, and this was not limited to patients with underlying medical conditions or predisposing healthcare-associated risk factors. This trend warrants further investigation into other potential community-acquired risk factors in this patient group such as travel and close contacts. A study by Kennedy and Collignon shows that approximately 50% of Australian travelers returning from high incidence countries such as India and China were colonised with drug-resistant *E. coli* and can remain colonised for months [20]. As such, specific questioning about travel is now recommended for all patients with presumed infections at ED admission, with modification of empiric antibiotic prescribing if positive. Due to the retrospective nature of our study, these risk factors could not be evaluated.

We observed a high rate of community-onset MRSA bacteraemia at 20%-30% throughout the 10-year study period. A nationwide surveillance of staphylococcal bacteraemia in Australia and New Zealand revealed that MRSA bacteraemia was no longer confined to hospitals and has become an issue in the community [2]. The incidence of MRSA bacteraemia in the ED setting, however, has been less clear. Rather than being the traditional community phenotype of MRSA (nmMRSA), the majority of our MRSA infections (57%) were of a HCA phenotype (multi-resistant) in patients with healthcare-associated risk factors. These risk factors appear to be an important guide to adding empiric anti-MRSA cover in those with presumed staphylococcal bacteraemia.

HCA BSI criteria were initially proposed by Friedman at el to help clinicians identify patients with a higher risk of acquiring MDR pathogen [3]. Subsequently, this concept has been consolidated further with the introduction of the American Thoracic Society and the Infectious Diseases Society of America guidelines, which suggest use of broad-spectrum antibiotics for those who meet the HCA criteria for healthcare-associated pneumonia [21]. Interestingly, in our study, 40% of those with COBSI caused by a susceptible organism (72/180) had a HCA risk factor (only two of these were infected with *P. aeruginosa*). This suggests that further studies are required to refine the risk factors for an infection caused by a MDR organism presenting to the ED in order to prevent overuse of broad-spectrum antibiotics.

The impact of antibiotic resistance on treatment and patient outcomes has varied considerably between studies [22-24]. One of the most consistent findings however is that infection with a MDR organism leads to an increase in receipt of inactive empiric antibiotic therapy [25,26]. Our data showed an association between inactive empiric therapy and 30-day mortality but this was not statistically significant after controlling for other confounders. The adverse consequences of inactive empiric therapy on increasing mortality in BSI remain inconsistent in the literature, especially for gram-negative bacteria, and depend on the source and severity of bacteraemia [27,28]. In our study, the high risk source of COBSI and higher Pitt bacteraemia score were shown to be independent predictors for mortality. However, severe sepsis/septic shock were not independent predictors and this was due to the fact that these covariates were captured within the Pitt bacteraemia score (correlation coefficient was > 0.7, Table 4). Although the presence of MDR organisms in COBSI was not associated with increased 30-day mortality, it was associated with an increase in length of hospital stay. Importantly, the economic impact of infections associated with MDR organisms originating from the community warrants further investigation to facilitate healthcare resource allocation.

The current study has several limitations. Due to its retrospective nature, risk factors such as recent travel, history of colonisation or close contacts with MDR organisms could not be assessed. Furthermore, mechanisms of resistance such as the presence of extended-spectrum β-lactamases or carbapenemases were not elucidated. An automated system (Vitek®) was used for susceptibility testing and therefore exact minimum inhibitory concentrations were not reported. This study was conducted at a large tertiary ED in southern Australia, and thus the results may not be generalisable to other EDs with different patient demographic profiles or clinical burden.

## Conclusions

This work has highlighted concerning trends toward greater antimicrobial resistance in common pathogens causing COBSI. An ideal model to predict COBSI with MDR organisms may incorporate a number of clinical factors (e.g. prior exposure of antibiotics, underlying immunosuppresion), relevant healthcare-associated risk factors, and possibly organism-specific risk factors (e.g. recent travel and MDR Enterobacteriaceae). These studies are essential to assist with the challenges of empiric antibiotic prescribing for those presenting to hospitals with suspected sepsis.

## Abbreviations

MDR: Multidrug-resistant; CO: Community-onset; BSI: Bloodstream infections; MRSA: Methicillin-resistant *Staphylococcus aureus*; GNB: Gram-negative bacilli; VRE: Vancomycin-resistant enterococci; ED: Emergency department; HCA: Healthcare-associated; LTCF: Long-term care facility; IV: Intravenous.

## Competing interests

DCMK has sat on advisory boards for Merck Sharp & Dohme (MSD), and Pfizer, and receives financial/travel support (not related to the current work) from Pfizer, Roche, Novartis, MSD and Gilead Sciences. AYP has been to one advisory board meeting for Abbott Molecular, Ortho-McNeil-Janssen, Pfizer and AstraZeneca, and has received a speaker’s honorarium from AstraZeneca and MSD for one presentation each. Other authors: none declared.

## Authors’ contributions

CJL contributed to the conception and design of the study, conducted data collection and interpretation, performed the statistical analysis and drafted the manuscript. ACC, DCMK and AYP were part of the conception of the study and participated in refining the study design, data interpretation and providing statistical advices, as well as revising the manuscript. AYP was also involved in critically appraising the manuscript and taking responsibility for the paper as a whole. All authors read and approved the final manuscript.

## Pre-publication history

The pre-publication history for this paper can be accessed here:

http://www.biomedcentral.com/1471-2334/14/126/prepub

## References

[B1] Pop-Vicas A Tacconelli E Gravenstein S Lu B D’Agata EM Influx of multidrug-resistant, Gram-negative bacteria in the hospital setting and the role of elderly patients with bacterial bloodstream infection Infect Control Hosp Epidemiol 2009 14 325 331 10.1086/596608 19220162 PMC5753794

[B2] Turnidge J Kotsanas D Munckhof W Roberts S Bennett C Nimmo G Coombs G Murray R Howden B Johnson PDR Dowling K Staphylococcus aureus bacteraemia: a major cause of mortality in Australia and New Zealand Med J Aust 2009 14 368 373 19807625 10.5694/j.1326-5377.2009.tb02841.x

[B3] Friedman ND Kaye KS Stout JE McGarry SA Trivette SL Briggs JP Lamm W Clark C MacFarquhar J Walton AL Reller LB Sexton DJ Health care-associated bloodstream infections in adults: a reason to change the accepted definition of community-acquired infections Ann Intern Med 2002 14 791 797 10.7326/0003-4819-137-10-200211190-00007 12435215

[B4] Garcia-Vidal C Viasus D Roset A Adamuz J Verdaguer R Dorca J Gudiol F Carratalà J Low incidence of multidrug-resistant organisms in patients with healthcare-associated pneumonia requiring hospitalization Clin Microbiol Infect 2011 14 1659 1665 10.1111/j.1469-0691.2011.03484.x 21463391

[B5] Aliberti S Di Pasquale M Zanaboni AM Cosentini R Brambilla AM Seghezzi S Tarsia P Mantero M Blasi F Stratifying risk factors for multidrug-resistant pathogens in hospitalized patients coming from the community with pneumonia Clin Infect Dis 2012 14 470 478 10.1093/cid/cir840 22109954

[B6] Schreiber MP Chan CM Shorr AF Resistant pathogens in nonnosocomial pneumonia and respiratory failure: is it time to refine the definition of health-care-associated pneumonia? Chest 2010 14 1283 1288 10.1378/chest.09-2434 20154075

[B7] Shorr AF Zilberberg MD Reichley R Kan J Hoban A Hoffman J Micek ST Kollef MH Validation of a clinical score for assessing the risk of resistant pathogens in patients with pneumonia presenting to the emergency department Clin Infect Dis 2012 14 193 198 10.1093/cid/cir813 22109951

[B8] Andrews JM BSAC Working Party on Susceptibility Testing BSAC standardized disc susceptibility testing method (version 8) J Antimicrob Chemother 2009 14 454 489 10.1093/jac/dkp244 19587067

[B9] European Committee on Antimicrobial Susceptibility Testing (EUCAST) EUCAST breakpoint table version 1.1 RNA 2010 [http://www.eucast.org]

[B10] O’Fallon E Pop-Vicas A D’Agata E The emerging threat of multidrug-resistant Gram-negative organisms in long-term care facilities J Gerontol A Biol Sci Med Sci 2009 14 138 141 19164271 10.1093/gerona/gln020 PMC2691192

[B11] Pop-Vicas AE Mitchell SL Kandel R Schreiber R D’Agata EMC Multidrug-resistant Gram-negative bacteria in a long-term care facility: prevalence and risk factors J Am Geriatr Soc 2008 14 1276 1280 10.1111/j.1532-5415.2008.01787.x 18557965

[B12] Turnidge JD Nimmo GR Pearson J Gottlieb T Collignon PJ Australian Group on Antimicrobial Resistance Epidemiology and outcomes for staphylococcus aureus bacteraemia in Australian hospitals, 2005–06: report from the Australian group on antimicrobial resistance Commun Dis Intell Q Rep 2007 14 398 403 18268881 10.33321/cdi.2007.31.43

[B13] Blot S Vandewoude K De Bacquer D Colardyn F Nosocomial bacteremia caused by antibiotic-resistant Gram-negative bacteria in critically ill patients: clinical outcome and length of hospitalization Clin Infect Dis 2002 14 1600 1606 10.1086/340616 12032895

[B14] Charlson ME Pompei P Ales KL MacKenzie CR A new method of classifying prognostic comorbidity in longitudinal studies: development and validation J Chronic Dis 1987 14 373 383 10.1016/0021-9681(87)90171-8 3558716

[B15] Ben-Ami R Schwaber MJ Navon-Venezia S Schwartz D Giladi M Chmelnitsky I Leavitt A Carmeli Y Influx of extended-spectrum β-lactamase-producing enterobacteriaceae into the hospital Clin Infect Dis 2006 14 925 934 10.1086/500936 16511754

[B16] Pop-Vicas AE D’Agata EM The rising influx of multidrug-resistant Gram-negative bacilli into a tertiary care hospital Clin Infect Dis 2005 14 1792 1798 10.1086/430314 15909268

[B17] Turnidge J Gottlieb T Mitchell D Pearson J Bell J Australian Group on Antimicrobial Resistance: Gram-negative Survey Community-onset Infections 2010 Antimicrobial Susceptibility Report RNA [http://www.agargroup.org/files/AGAR%20GNB10%20REAL%20FINAL.pdf]

[B18] Antibiotic Expert Group Therapeutic guidelines: antibiotic, version 14 2010 Melbourne: Therapeutic Guidelines Limited

[B19] Colodner R Rock W Chazan B Keller N Guy N Sakran W Raz R Risk factors for the development of extended-spectrum beta-lactamase-producing bacteria in nonhospitalized patients Eur J Clin Microbiol Infect Dis 2004 14 163 167 10.1007/s10096-003-1084-2 14986159

[B20] Kennedy K Collignon P Colonisation with escherichia coli resistant to “critically important” antibiotics: a high risk for international travellers Eur J Clin Microbiol Infect Dis 2010 14 1501 1506 10.1007/s10096-010-1031-y 20835879

[B21] American Thoracic Society; Infectious Diseases Society of America Guidelines for the management of adults with hospital-acquired, ventilator-associated, and healthcare-associated pneumonia Am J Respir Crit Care Med 2005 14 388 416 15699079 10.1164/rccm.200405-644ST

[B22] de Kraker ME Wolkewitz M Davey PG Koller W Berger J Nagler J Icket C Kalenic S Horvatic J Seifert H Kaasch A Paniara O Argyropoulou A Bompola M Smyth E Skally M Raglio A Dumpis U Melbarde Kelmere A Borg M Xuereb D Ghita MC Noble M Kolman J Grabljevec S Turner D Lansbury L Grundmann H Burden of antimicrobial resistance in European hospitals: excess mortality and length of hospital stay associated with bloodstream infections due to escherichia coli resistant to third-generation cephalosporins J Antimicrob Chemother 2011 14 398 407 10.1093/jac/dkq412 21106563

[B23] Liao CH Chen SY Huang YT Hsueh PR Outcome of patients with meticillin-resistant Staphylococcus aureus bacteraemia at an emergency department of a medical centre in Taiwan Int J Antimicrob Agents 2008 14 326 332 10.1016/j.ijantimicag.2008.04.011 18617372 PMC7127075

[B24] Schwaber MJ Navon-Venezia S Kaye KS Ben-Ami R Schwartz D Carmeli Y Clinical and economic impact of bacteremia with extended-spectrum-beta-lactamase-producing Enterobacteriaceae Antimicrob Agents Chemother 2006 14 1257 1262 10.1128/AAC.50.4.1257-1262.2006 16569837 PMC1426954

[B25] Peralta G Snchez MB Garrido JC De-Benito I Cano ME Martnez-Martnez L Roiz MP Impact of antibiotic resistance and of adequate empirical antibiotic treatment in the prognosis of patients with Escherichia coli bacteraemia J Antimicrob Chemother 2007 14 855 863 10.1093/jac/dkm279 17644532

[B26] Rodríguez-Baño J Picón E Gijón P Hernández José R Ruíz M Peña C Almela M Almirante B Grill F Colomina J Giménez M Oliver A Horcajada Juan P Navarro G Coloma A Pascual A Community-onset bacteremia due to extended-spectrum β-lactamase-producing escherichia coli: risk factors and prognosis Clin Infect Dis 2010 14 40 48 10.1086/649537 19995215

[B27] Kang CI Kim SH Park W Lee KD Kim HB Kim EC Oh MD Choe KW Bloodstream infections caused by antibiotic-resistant gram-negative bacilli: risk factors for mortality and impact of inappropriate initial antimicrobial therapy on outcome Antimicrob Agents Chemother 2005 14 760 766 10.1128/AAC.49.2.760-766.2005 15673761 PMC547233

[B28] Lee YT Kuo SC Yang SP Lin YT Tseng FC Chen TL Fung CP Impact of appropriate antimicrobial therapy on mortality associated with acinetobacter baumannii bacteremia: relation to severity of infection Clin Infect Dis 2012 14 209 215 10.1093/cid/cis385 22495546

